# Correlation between acromiohumeral distance and the severity of supraspinatus tendon tear by ultrasound imaging in a Chinese population

**DOI:** 10.1186/s12891-020-3109-8

**Published:** 2020-02-17

**Authors:** Mingmin Xu, Zhenping Li, Youfa Zhou, Bin Ji, Suming Tian, Gang Chen

**Affiliations:** 1 Department of Ultrasound, The First Affiliated Hospital of Jiaxing College, The First Hospital of Jiaxing City, Jiaxing City, China; 2 Department of Anesthesiology, Sir Run Run Shaw Hospital, School of Medicine, Zhejiang University, No. 3 Qingchun East Road, Hangzhou, 310016 China; 3 Department of Anesthesiology, The First Affiliated Hospital of Jiaxing College, The First Hospital of Jiaxing City, Jiaxing City, China; 4 Department of Orthopedics, The First Affiliated Hospital of Jiaxing College, The First Hospital of Jiaxing City, Jiaxing City, China

**Keywords:** Acromiohumeral distance, Supraspinatus tendon, Shoulder, Ultrasound

## Abstract

**Background:**

The aim of this study was twofold: (i) to investigate the intrarater reliability of acromiohumeral distance measurement; (ii) to assess the level of association between acromiohumeral distance measured by ultrasonography, and the degree of supraspinatus tendon tear, in patients suffering from chronic shoulder pain.

**Methods:**

A cross-sectional, case-control study was carried out. A convenience sample comprising 59 patients with a unilateral supraspinatus tendon tear was assessed. Both shoulders of each patient were scanned by ultrasound, with the contralateral asymptomatic shoulders serving as the control group for comparison. Acromiohumeral distances of each shoulder were measured and analysed.

**Results:**

Intrarater reliability was excellent for the ultrasound method of acromiohumeral distance measurement. The acromiohumeral distance of shoulders with full-thickness supraspinatus tendon tear was significantly smaller than that of joints with partial-thickness supraspinatus tendon tear and an intact supraspinatus tendon. There was a significant positive correlation between reduced acromiohumeral distance and the severity of a supraspinatus tendon tear.

**Conclusions:**

Ultrasound is a reliable tool to measure acromiohumeral distance. A positive relationship was found between a narrowed acromiohumeral distance and the severity grading of a supraspinatus tendon tear. Reduced acromiohumeral distance can be considered a predictive parameter for a full-thickness supraspinatus tendon tear.

**Trial registration:**

The study was prospectively registered with the Chinese Clinical Trial Registry. Registration number: ChiCTR-ROC-17013550. Date of registry: 26 November 2017.

## Background

Rotator cuff tear (RCT) is one of the primary disorders of the shoulder [1]. The consequences of RCT are pain and functional loss. Whether RCT is caused by degeneration, or by extrinsic mechanical compression, is still under debate [2, 3]. Narrowing of the subacromial space can lead to impingement, which is considered to be the cause of rotator cuff tear progression [4, 5]. However, Michener found that the acromiohumeral space was not narrowed in patients with impingement syndrome [6].

Surgical interventions for RCT are based on theorised mechanisms [7]. Narrowing of the subacromial space is a predictor of the likelihood of a successful outcome after the rotator cuff repair is reduced [4, 8]. An unfavorable outcome in a patient with a small subacromial space can be explained by the association of a short acromiohumeral distance (AHD) with a large rotator cuff tear [9]. The subacromial space is quantified by the AHD. It is therefore essential that a reliable method of AHD measurement is identified [4]. Previously, AHD has been studied through a standard X-ray of the shoulder [9, 10]. However, radiographic AHD measurement is problematic as it is affected by both the patient’s position and X-ray beam direction [11]. On the other hand, ultrasound (US) has shown excellent reliability in AHD measurement in recent research [12].

The supraspinatus tendon (ST), which runs through the subacromial space, is most commonly affected in RCT [13, 14]. A narrowed AHD and the severity of supraspinatus tendon tear (STT) can be used as important criteria for surgical decision making in ST repair (repair plus acromioplasty vs. repair only) [15]. However, whether narrowing of the AHD is related to the severity of STT is not well known. Moreover, few AHD ultrasound imaging studies have been focused on the Chinese population. The purpose of this study was to confirm the reliability of the US method for AHD measurement and to evaluate the relationship between the narrowing of the AHD and the severity of STT.

## Methods

### Procedure

This study was a cross-sectional, case-control design. From December 2017 to December 2018, a convenience sample of 71 non-athlete patients with unilateral chronic shoulder pain (more than three months) and a limited motion was recruited from the Orthopedic inpatient ward of the primary investigator. Patients were firstly recruited to the study, then assessed for eligibility. Finally, bilateral shoulders of all patients were arranged for ultrasound and arthroscopy in order of precedence. The inclusion criteria were: (i) acquisition of US imaging (including measuring AHD and diagnosing the presence or absence of a tear on the ST) of the shoulder with the arm in a neutral position; (ii) shoulder arthroscopy carried out no later than 1 week after US imaging at our institution according to a standardized protocol; (iii) unilateral STT confirmed by US imaging and shoulder arthroscopy. Participants meeting all inclusion criteria were studied. Twelve participants did not satisfy these inclusion criteria. Therefore the final sample comprised 59 participants who were enrolled and divided into three groups. The full-thickness STT group (FG) included 28 adults, and the partial-thickness STT group (PG) included 31 patients. The contralateral asymptomatic shoulders with intact STT identified by US imaging were compared as the control group (CG). Exclusion criteria included previous surgery, fractures around the shoulder joint, arthritis, osteonecrosis, infection, acromioclavicular joint dislocation, shoulder tumour, a history of shoulder radiation therapy, congenital shoulder anomalies, shoulder glenohumeral instability, and contraindications to shoulder surgery. The studied population was Chinese and exclusively Asian. All participants provided written informed consent, and the study was approved by the Medical Ethics Committee of The First Hospital of Jiaxing city (No.2017089). All of the procedures were performed in accordance with the Declaration of Helsinki and relevant policies in China.

### Outcome measures

#### Acromiohumeral distance (AHD)

**Fig. 1:**
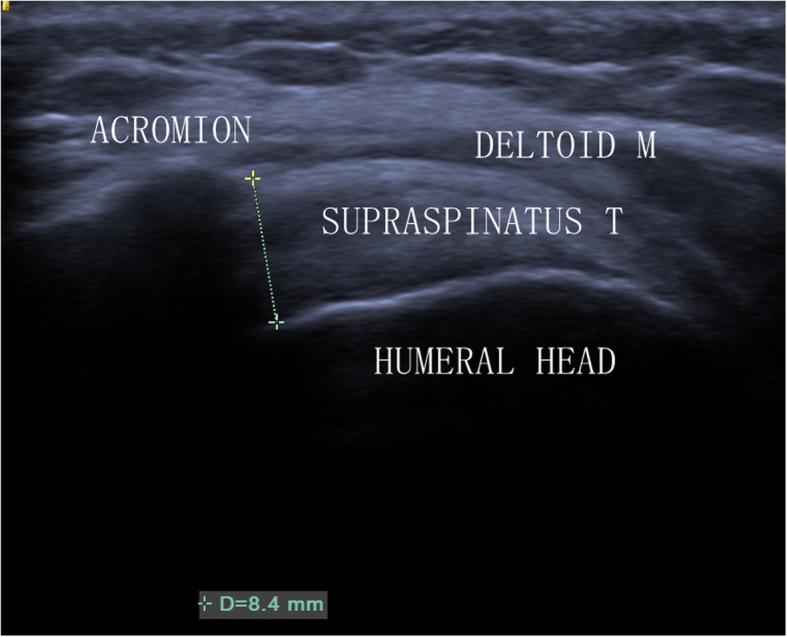
Sonogram of AHD in a normal left shoulder. AHD = 8.4 mm. T = tendon; M = muscle

Sonographic examination was conducted by US (Siemens ACUSON S3000, Siemens Medical Solutions, Mountain View, CA, USA). The patient was seated, with the upper limb in a neutral position. A 5–12 MHz linear transducer was placed in the coronal plane over the anterior aspect of the acromion (Fig. 1). AHD was defined as the shortest distance between the acromion and the humeral head [16]. Both shoulders of each patient were imaged by a single examiner, who had ten years of musculoskeletal ultrasound experience. Two measurements repeatedly on each side of the same shoulders were taken at an interval of 1 min. The ultrasound examiner was blind to all measurements (values were obscured by placing a sticker on the ultrasound screen). A research assistant took the measurements and entered them into a database [17]. Sonographic AHD measurements were taken in millimetres.

#### Data analysis

Quantitative variables are expressed as the mean (standard deviation (SD)). Qualitative variables are described as numbers and percentages. Comparison between groups was carried out with the student’s *t-*test for quantitative variables and the chi-square test for qualitative variables when necessary. The intra-observer correlation coefficient (ICC) was calculated by making measurements twice on each side of the same shoulders to evaluate the consistency of AHD measured by US, and the average values of the two measurements were used for the final statistical tests between groups. An ICC value less than 0.50 was an indication of “poor” reliability; “moderate” between 0.50 and 0.75, “good” between 0.76 and 0.90; and excellent over 0.90 [17]. One way ANOVA was used to compare the AHD values among the three research groups. AHD differences were analysed by Bonferroni’s post-hoc tests when significant differences were found in the ANOVA. The Spearman correlation coefficient was used to assess the association between AHD and the severity of STT. We considered r values < 0.3 to represent a weak association, 0.3–0.7 to represent a moderate association, and > 0.7 to express a strong association [18]. *P* < 0.05 was considered statistically significant. All analyses were performed using SPSS versions 19.0 (IBM, Armonk, NY, USA).

## Results

According to the inclusion and exclusion criteria mentioned above, 59 patients with unilateral STT who underwent arthroscopy and were retrospectively analysed in this study. Arthroscopy confirmed full-thickness STT in 28 cases, and partial-thickness STT in 31 cases.

**Fig. 2:**
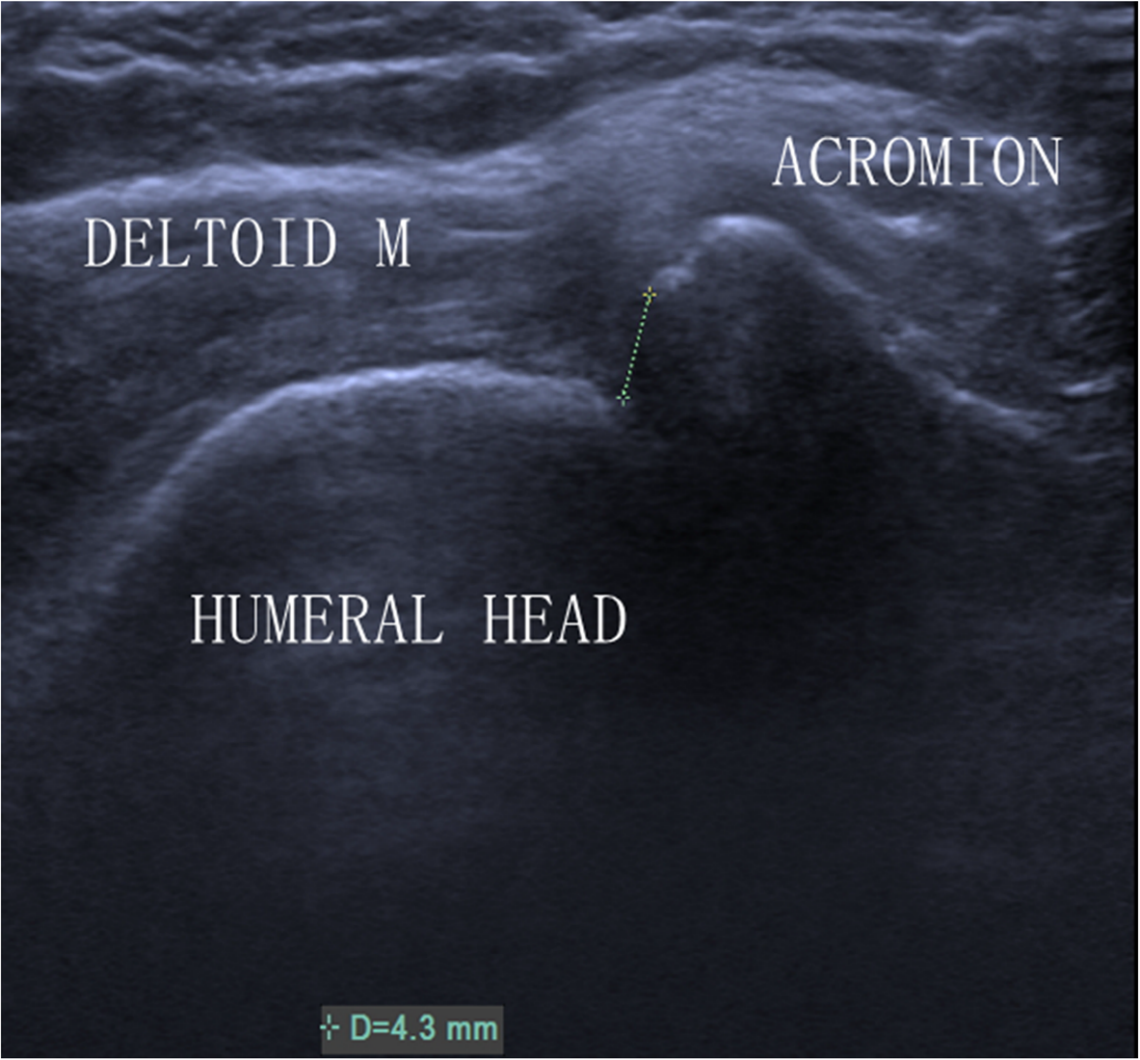
Ultrasound image of AHD in a shoulder with a full-thickness tear of the supraspinatus tendon. M = muscle

**Fig. 3:**
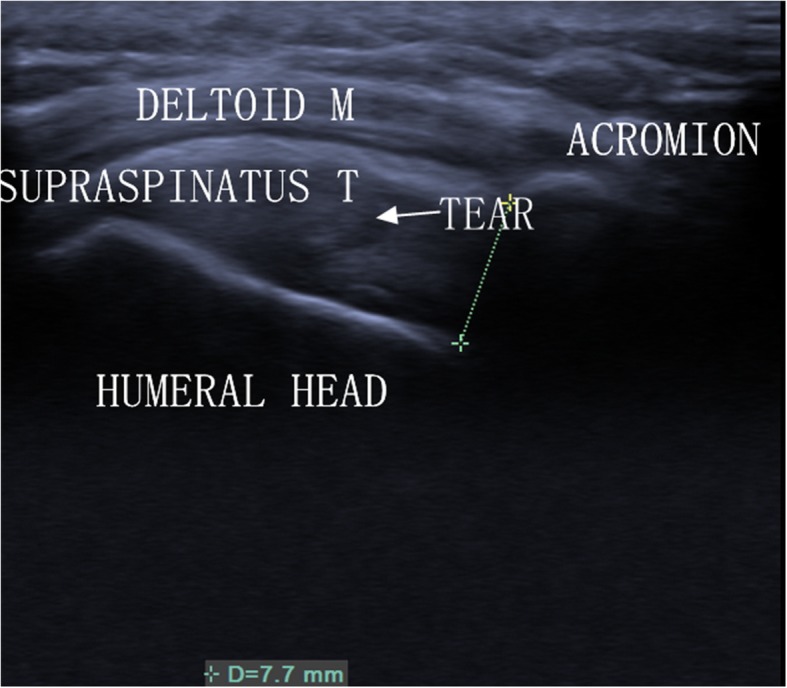
Ultrasound image of AHD in a shoulder with a partial-thickness tear of the supraspinatus tendon. T = tendon; M = muscle

Analysis of the data revealed three key points: (1) The ICC was 0.98 (0.96–0.99) for intra-observer reproducibility. (2) In the FG, US measured a reduced AHD (Fig. 2). (3) In the PG, AHD measured by US was normal (Fig. 3). Demographic data of the 59 patients were summarised in Table 1.

**Table 1:** Sample characteristics

Parameter	FG	PG	*p*-value
No.	28	31	
Age (years)			0.752
Mean (SD)	54.5 (10.5)	53.6 (11.2)	
Gender			0.625
Male	10	13	
Female	18	18	
Right-handed	28	31	
Dominance			0.811
Dominant side	20	23	
Nondominant side	8	8	

*FG* Full-thickness supraspinatus tendon tear group

*PG* Partial-thickness supraspinatus tendon tear group

*p*: statistical significance between the two groups. A *p*-value of less than 0.05 was considered significant

**Fig. 4:**
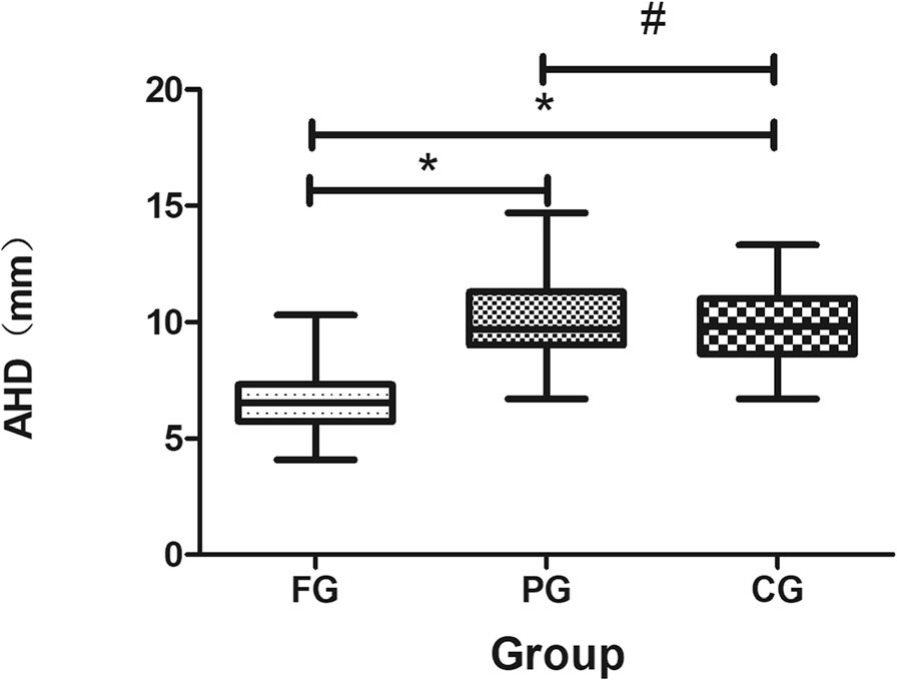
Boxplot comparing the AHD in FG, PG, and CG. *P < 0.001, ^#^*P>*0.05

One-way ANOVA analysis of AHD showed a difference among the three groups (Table 2). Further analysis by Bonferroni’s post-hoc tests demonstrated a significantly reduced AHD (6.6 mm (SD 1.4) in the FG. However, no significant difference was found between the AHDs of the PG and the CG (Fig. 4)*.*

**Table 2:** One-way ANOVA analysis of AHD among the three groups

Parameter	AHD (mm) Mean (SD)	*F*	*p*
FG	6.6 (1.4)		
PG	10.0 (1.9)		
CG	9.8 (1.6)		
		38.006	0.000

*FG* Full-thickness supraspinatus tendon tear group

*PG* Partial-thickness supraspinatus tendon tear group

*CG* Control group

*F* represents One-way ANOVA analysis of AHD among the three groups above

*p*: statistical significance among the three groups. The significance level was set at *p* < 0.05

The Spearman correlation coefficient showed a statistical difference in the AHD between the FG and the CG (*P* = 0.000). However, no statistical difference was found between the AHDs of the PG and the CG (*P* = 0.800) (Table 3).

**Table 3:** Spearman correlation coefficient analysis for AHD

Parameter	AHD (mm) Mean (SD)	*r*	*p*
FG	6.6 (1.4)	0.729^a^	<0.001
PG	10.0 (1.9)	0.027^b^	0.800
CG	9.8 (1.6)		

*FG* Full-thickness supraspinatus tendon tear group

*PG* Partial-thickness supraspinatus tendon tear group

*CG* Control group

*r*^a^ represents the correlation coefficient between FG and CG

*r*^b^ represents the correlation coefficient between PG and CG

*P* < 0.05 was considered statistically significant

## Discussion

In this study, we confirmed that intra-rater reliability was excellent for the US method of AHD measurement, supporting the findings of previous studies [5, 12].

Previously, AHD has been studied in literature mainly through standard X-ray of the shoulder [9, 10]. However, the reliability of radiological AHD measurement has not been supported by a review of studies to date [4]. Our results show excellent reliability of AHD measurement by US. Furthermore, US and magnetic resonance imaging had comparable accuracy for diagnosing an RCT [19], which gave us confidence that the US technique could be used in place of the radiographic technique for clinical purposes.

Due to studies having been carried out in different populations, with either healthy participants or participants with rotator cuff tendinopathy of different subtypes, the reduction of AHD as a mechanism in the aetiology of RCT is controversial [3, 6, 20]. In our study, the AHD findings in 59 non-athlete patients revealed a significantly smaller AHD in FG (*P* < 0.001). A positive relationship was found between a reduced AHD and the severity of STT [21]. Saupe also found a higher prevalence of full-thickness STT in the reduced AHD patient group [9]. Thus, a reduced AHD can be considered as a predictive parameter for a full-thickness STT. However, this method of measurement cannot differentiate between an intact ST and partial-thickness STT, which indicates that reduced AHD, as identified by US, cannot be used as a single criterion for surgical decision making in rotator cuff repair [9].

Possible explanations for the reduction of AHD include superior translation of the humeral head due to increased deltoid activation and biceps dislocation, or infraspinatus muscular fatty degeneration and atrophy. In these cases, surgical repair might be questionable [22]. The AHD value was more prognostic than diagnostic.

Previous in-vivo studies had reported that AHD ranges from approximately 2 to 17 mm. This wide range of AHD measurements reflects differences in race, age, gender, shoulder position, shoulder pathology, and the measurement technique. It had also been reported that muscle activity (in particular, adducting and abducting muscle activity) had a significant effect on AHD [23–25].

There was a higher STT occurrence in females than males in this study, with the prevalence of STT being greater on the dominant shoulder [5]. It is not clear if the differences in AHD between the two sides represents accommodation due to overuse or the participants’ side dominance.

There are several limitations to this study. Firstly, in contrast to X-ray and MRI [9], the ultrasound method did not measure potential AHD, since it does not allow the measurement of the inferior protrusion of the acromioclavicular joint, as the penetration of beams to this area is not possible [5]. Secondly, this material pooled conclusions from abnormal shoulders without taking into account age, tear size, atrophy and muscle degeneration, or coracoacromial ligament and scapular morphology [26, 27]. Thirdly, this study did not take into account the tear of the infraspinatus and subscapularis tendons. Finally, the current study measured AHD with the arm positioned at rest. Further investigation of AHD with active arm elevation in patients with STT is necessary.

## Conclusions

Ultrasound is a reliable tool to measure acromiohumeral distance. This study identified differences in AHD between individuals with STT of different subtypes using US. A positive relationship was found between the narrowing of AHD and the severity of an STT. A reduced AHD can be considered as a predictive parameter for a full-thickness STT.

## Data Availability

The datasets used and analysed during the current study are available from the corresponding author upon reasonable request.

## Abbreviations

AHD: Acromiohumeral distance
ANOVA: Analysis of variance
CG: Control group
FG: Full-thickness supraspinatus tendon tear group
ICC: Intraobserver correlation coefficient
PG: Partial-thickness supraspinatus tendon tear group
RCT: Rotator cuff tear
SD: Standard deviation
ST: Supraspinatus tendon
STT: Supraspinatus tendon tear
US: Ultrasound
